# TRPV6 modulates proliferation of human pancreatic neuroendocrine BON-1 tumour cells

**DOI:** 10.1042/BSR20160106

**Published:** 2016-08-24

**Authors:** Marek Skrzypski, Paweł A. Kołodziejski, Stefan Mergler, Noushafarin Khajavi, Krzysztof W. Nowak, Mathias Z. Strowski

**Affiliations:** * Department of Animal Physiology and Biochemistry, Poznań University of Life Sciences, 60-637 Poznań, Poland; † Department of Hepatology and Gastroenterology and Interdisciplinary Centre of Metabolism: Endocrinology, Diabetes and Metabolism, Charité-University Medicine Berlin, 13353 Berlin, Germany; ‡ Department of Ophthalmology, Charité-University Medicine Berlin, 13353 Berlin, Germany; § Institute for Experimental Pediatric Endocrinology, Charité-University Medicine Berlin, 13353 Berlin, Germany; ║ Medical Clinic 1, Department of Gastroenterology, Elblandklinik, 01662 Meissen, Germany

**Keywords:** BON-1, calcium, neuroendocrine tumour, proliferation, QGP-1, TRPV6

## Abstract

Highly Ca^2+^ permeable receptor potential channel vanilloid type 6 (TRPV6) modulates a variety of biological functions including calcium-dependent cell growth and apoptosis. So far, the role of TRPV6 in controlling growth of pancreatic neuroendocrine tumour (NET) cells is unknown. In the present study, we characterize the expression of TRPV6 in pancreatic BON-1 and QGP-1 NET cells. Furthermore, we evaluate the impact of TRPV6 on intracellular calcium, the activity of nuclear factor of activated T-cells (NFAT) and proliferation of BON-1 cells. TRPV6 expression was assessed by real-time PCR and Western blot. TRPV6 mRNA expression and protein production were down-regulated by siRNA. Changes in intracellular calcium levels were detected by fluorescence calcium imaging (fura-2/AM). NFAT activity was studied by NFAT reporter assay; cell proliferation by bromodeoxyuridine (BrdU), MTT and propidium iodine staining. TRPV6 mRNA and protein are present in BON-1 and QGP-1 NET-cells. Down-regulation of TRPV6 attenuates BON-1 cell proliferation. TRPV6 down-regulation is associated with decreased Ca^2+^ response pattern and reduced NFAT activity. In conclusion, TRPV6 is expressed in pancreatic NETs and modulates cell proliferation via Ca^2+^-dependent mechanism, which is accompanied by NFAT activation.

## INTRODUCTION

The transient receptor potential (TRP) channel superfamily is a large group of ion channels composed of seven subgroups [1]. One of them is the TRP vanilloid family (TRPV), which consists of six ion channels. TRPVs modulate a variety of cellular processes including hormone secretion, cell migration as well as growth and apoptosis [1,2]. Numerous studies collectively indicated that these effects are predominantly mediated by calcium-dependent mechanisms [3–6]. Among all known TRPV channels, TRPV6 has the highest selectivity for Ca^2+^ ions [7]. TRPV6 was initially identified in rat duodenum, where it was implicated in regulating calcium absorption in enterocytes [8]. Later studies showed that TRPV6 is overexpressed in prostate and breast cancer cells, where it was relevant at stimulating calcium-dependently cancer cell proliferation and survival [6,9–11]. Consistently, down-regulation of TRPV6 in prostate cancer cells resulted in decreased cancer cell growth and higher cell death rate [6,12]. Thus, TRPV6 is currently suggested as a novel target in prostate cancer therapy. However, in oesophageal squamous cell carcinoma TRPV6 is down-regulated, which is associated with a poor survival in male patients [13]. Therefore it appears that TRPV6 function may be cancer cell type specific.

Little is known about the expression and the role of TRPV6 in pancreatic neoplasms. TRPV6 is present in pancreatic adenocarcinoma and insulinoma cells [14,15]. In insulin-producing INS-1E cells, TRPV6 down-regulation impairs cell growth and inhibits insulin mRNA expression [15]. However, studies addressing the expression and function of TRPV6 in pancreatic neuroendocrine tumour cells (NETs) are not yet available.

In the present study, we investigated the expression of TRPV6 in human pancreatic NET cells using well-established human BON-1 and QGP-1 cell lines [16,17]. Furthermore, we studied the role of this channel in controlling calcium homoeostasis and proliferation of BON-1 NET cells. Since nuclear factor of activated T-cells (NFAT) was recently reported to confer promitogenic role of TRPV6 in prostate cancer cells [6], we also studied NFAT expression relationship between TRPV6 and NFAT activity in NET cells.

## MATERIALS AND METHODS

### Materials

All cell culture media and supplements were purchased from Biochrom AG. Unless otherwise stated, all other reagents were from Sigma–Aldrich. Primary rabbit anti-TRPV6 antibody was purchased from Santa Cruz Biotechnology. Mouse β-actin and all secondary antibodies were purchased from Sigma–Aldrich.

### Cell culture

BON-1 cells were from Dr Courtney M. Townsend, Jr. (University of Texas Medical Branch, Texas, USA). QGP-1 cells were from Japanese Health Sciences Foundation, Osaka, Japan. BON-1 cells were cultured in DMEM/Ham's F12, QGP-1 cells and LCC-18 in RPMI medium at 37°C in a humidified atmosphere (5% CO_2_, 95% air). All experiments were performed in medium containing 10% FBS, 100 kU/l penicillin and 100 mg/l streptomycin.

### siRNA transfection

BON-1 cells were transfected with siRNA using HiPerfect reagent (Qiagen), according to the manufacturer's protocol. ON-TARGETplus SMARTpool of four individual TRPV6 siRNAs or non-targeting (nt) siRNA were obtained from Thermo Scientific Dharmacon. In brief, before transfection BON-1 cells were seeded in culture dishes. For determination of cell proliferation using bromodeoxyuridine (BrdU) and MTT assays, cells were seeded in 96-well plates (1×10^4^ cells/well). For gene expression analysis, Western blot or cell cycle analysis, cells were seeded in 6-well plates (1.6×10^5^ cells/well). Thereafter nt or TRPV6 siRNA (both at the concentration of 30 nM) were used for fast-forward transfection. Cells were incubated in the presence of siRNA for 12 h. Suppression of TRPV6 mRNA expression and protein production by TRPV6 siRNA was monitored 24, 48 and 72 h after siRNA application.

### Real-time PCR

Total RNA was extracted using Tripure reagent (Roche Diagnostics). cDNA was generated from 1 μg of RNA using High capacity cDNA reverse transcription kit (Life Technologies). Real time PCR was performed on QuantStudio 12K Flex™ Real-Time PCR system (Life Technologies). PCR with gene specific primers (Supplementary Table S1) was performed by using Fast SYBR Green Master Mix. Relative gene expression was determined by ΔΔCT method. *GAPDH* (glyceraldehyde 3-phosphate dehydrogenase) was used as reference gene.

### Western blot

Proteins were isolated using RIPA buffer (25 mM Tris/HCl pH 7.6, 150 mM NaCl, 5 mM EDTA, 1% NP-40 or 1% Triton X-100, 1% sodium deoxycholate, 0.1% SDS) supplemented with protease inhibitor cocktail (Roche Diagnostics). Western blot signals obtained with TRPV6 or β-actin antibodies were quantified as previously described [18].

### Calcium imaging

The intracellular Ca^2+^ concentration in BON-1 cells was measured as previously described [4]. In brief, 2 days after nt or TRPV6 siRNA transfection, cells were pre-incubated with the fluorescent dye fura-2/AM (2 μM) for 30–40 min at 37°C. The fura-2 reaction was stopped with a Ringer-like (control) solution containing (mM): 150 NaCl, 6 CsCl, 1 MgCl_2_, 10 glucose, 10 HEPES and 1.5 CaCl_2_, pH of 7.4. Cells were then washed three times using the same solution to remove cell debris or dead cells. Fluorescence measurements were performed at room temperature using a microscope (Olympus BW50WI) connected to a digital imaging system (TILL Photonics) suited for UV excitation. TIDA software was used (HEKA Electronics). Fura-2/AM fluorescence was alternately excited at wavelengths of 340 and 380 nm and emission was measured at 510 nm. The fluorescence ratio (*f*340 nm/*f*380 nm) is a relative index of changes in [Ca^2+^]_i_ [19]. Prior the experiments, cells were routinely tested to determine whether the control baseline was constant for 8–20 min (results not shown). For each measurement, the constant basal levels of [Ca^2+^]_i_ were confirmed during the first 3 min, followed by an isoosmotic replacement with a Ca^2+^-free Ringer-like solution (1 mM EGTA). After 3 min, 1.5 mM Ca^2+^ was added to increase [Ca^2+^]_i_. The reversibility of Ca^2+^ changes is an indicator of cell viability and functional relevance of the Ca^2+^ sensing via Ca^2+^ channels such as TRPV6 [11,12,20]. Results are presented as mean traces of *f*340/*f*380 ± S.E.M.

### Determination of NFAT activity

The consequences of TRPV6 down-regulation in BON-1 cells on NFAT activity were assessed using NFAT reporter assay (Qiagen) 48 h after TRPV6 siRNA transfection, as previously described in our earlier study [15].

### Determination of cell proliferation

Cell proliferation was assessed using a Cell Proliferation ELISA BrdU colorimetric kit (Roche Diagnostics). In brief, BON-1 cells were seeded in 96-well plates and transfected with nt or TRPV6 siRNA. After 24, 48, or 72 h, BrdU solution (10 μM) was added and cells were incubated for 3 h. The amount of BrdU incorporation into DNA was determined according to manufacturer's instruction.

### Determination of cell viability

To determine viable cells, MTT assay was performed. Cells transfected either with nt or TRPV6 siRNA were analysed using MTT assay. MTT solution was added to the wells (0.5 mg/ml) 48 h after transfection of cells either with nt or TRPV6 siRNA. Then, cells were incubated with MTT for 3 h. Thereafter, medium was removed from wells and formazan crystals were dissolved in 150 μl DMSO. Absorbance of samples was measured at 570 and 650 nm wave lengths using Synergy 2 Multi-Mode Microplate Reader (BioTek).

### Cell cycle analysis

The consequences of TRPV6 down-regulation in BON-1 cells on cell cycle were determined using propidium iodide (PI) staining 48 h after siRNA transfection, as described [15].

### Statistical analysis

Data were analysed using ANOVA, followed by the Bonferroni test. *P*<0.05 (*), *P*<0.01 (**). The Student's *t* test (parametric two-tailed *t* test) was used for statistical significance determination between two sets of data. For the evaluation of calcium imaging experiments, significance was determined using Student's *t* test for paired and unpaired data (*P*-values: two-tailed) provided they passed a normality test according to Kolmogorov–Smirnov. If the normality test failed, non-parametric tests were used. Probabilities of *P*<0.05 [indicated by asterisks (*) and hash tags (#)] were considered to be significant. Results are shown as means ± S.E.M. and were derived in representative experiments performed in four or three (Western blot) replicates at least.

## RESULTS

### Expression of TRPV6 in NET cells

**Figure 1:**
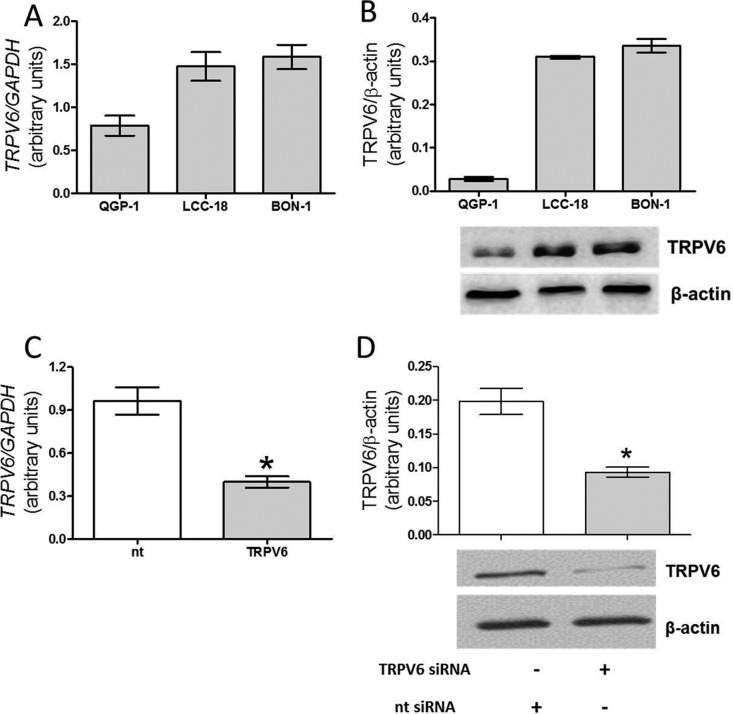
TRPV6 mRNA expression and protein production in NET cells (**A**) Real time PCR detection of TRPV6 mRNA expression in QGP-1, BON-1 and LCC-18 cells. (**B**) Western blot detection of TRPV6 protein in BON-1, QGP-1 and LCC-18 cells. (**C**) Suppression of TRPV6 mRNA expression in BON-1 cells transfected with siRNA for 48 h in comparison with BON-1 cells transfected with non-targeting construct (nt). (**D**) Suppression of TRPV6 protein production in BON-1 cells 48 h after siRNA transfection in comparison with nt BON-1 cells. Results are the mean ± S.E.M., obtained from at least *n*=3.

We detected TRPV6 mRNA and protein in all three different NET cell lines; pancreatic BON-1 and QGP-1 cells by real-time PCR as well as by Western blot (Figures 1A and 1B). Notably, also the colonic NET cells LCC-18 expressed TRPV6 at mRNA and protein levels (Figures 1A and 1B). The highest levels of TRPV6 mRNA expression and protein levels were found in BON-1 and LCC-18 cells. Taking into account the need of experimental suppression of TRPV6 in our study and due to a low expression of TRPV6 in QGP-1 cells, all subsequent experiments were performed in BON-1 cells. Transfection of BON-1 cells with TRPV6 siRNA for 48 h caused a suppression of mRNA expression by approximately 65% (Figure 1C), whereas protein production decreased by approximately 60%, as compared with nt siRNA transfected cells (Figure 1D).

### TRPV6 controls Ca^2+^ regulation in BON-1 cells

**Figure 2:**
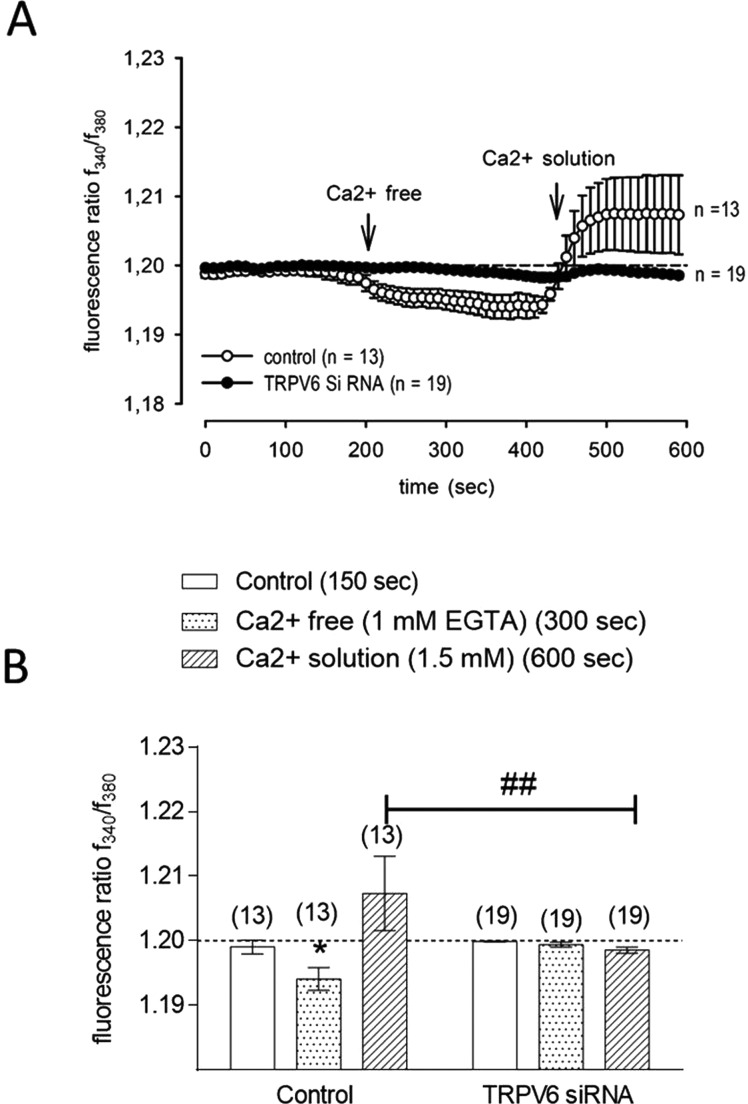
Effect of TRPV6 down-regulation on Ca^2+^ regulation in BON-1 cells (**A**) Measurement of intracellular Ca^2+^ concentration in cells transfected either with TRPV6 or nt siRNA. The basal Ca^2+^ level was measured during the first 200 s followed by Ca^2+^ reduction (left arrow) under Ca^2+^-free condition and a Ca^2+^ increase after 1.5 mM Ca^2+^ exposure at 380 s. (right arrow). Intracellular Ca^2+^ increases above the base line (dashed line) in nt siRNA-transfected cells could be detected after re-addition of extracellular Ca^2+^ (open circles) (*n*=13). In contrast, this effect could be clearly suppressed in TRPV6 siRNA-transfected BON-1 cells (filled circles) (*n*=19). Changes in cytosolic free Ca^2+^ are depicted as the ratio of the fluorescence induced by excitation wavelength of 340 and 380 nm by the equation of Grynkiewicz et al. [19]. (**B**) Summary of the experiments with nt and TRPV6 siRNA-transfected BON-1 cells. Statistical evaluation of [Ca^2+^]_i_ was performed after 300 s (light grey bars) and 600 s (dark grey bars).

To characterize the role of TRPV6 at controlling intracellular calcium accumulation in pancreatic BON-1 NET cells, we tested the responses of nt or TRPV6 siRNA transfected cells to rapid changes of intracellular Ca^2+^ concentration ([Ca^2+^]_i_) from a Ca^2+^-free to a 1.5 mM Ca^2+^-containing extracellular solution. In a Ca^2+^-free solution, the fluorescence ratio (*f*340/*f*380) corresponding to [Ca^2+^]_i_ decreased from 1.199±0.001 (150 s) to 1.194±0.001 (*n*=13; *P*<0.005; *t*=300 s) in nt siRNA-transfected BON-1 cells (Figures 2A and 2B). In the presence of 1.5 mM extracellular Ca^2+^, *f*340/*f*380 increased above the baseline (1.207±0.005; *n*=13; *t*=550 s). In cells with down-regulated TRPV6, no change in *f*340/*f*380 was detected in the Ca^2+^-free solution until 370 s and only a very slight decrease to 1.199±0.003 was recorded at 400 s (*n*=19). After replacement with the Ca^2+^ solution, the fluorescence ratio increased back to the baseline. Thus, changes of [Ca^2+^]_i_ in a Ca^2+^-free and a Ca^2+^ containing solution were completely inhibited in TRPV6 siRNA-transfected cells as compared with nt transfected BON-1 cells (*n*=19; *P*<0.01).

### TRPV6 modulates pancreatic BON-1 NET cell proliferation

**Figure 3:**
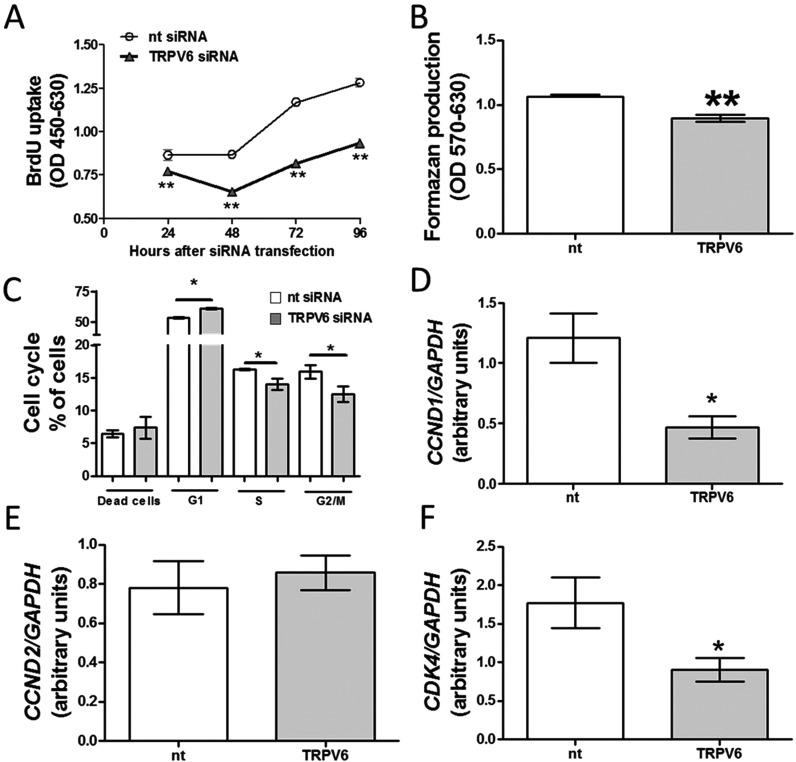
Effects of TRPV6 down-regulation on BON-1 cells proliferation and cell cycle (**A**) BON-1 cell proliferation assed 24, 48 and 72 h after transfection with siRNA. (**B**) The number of viable BON-1 cells studied 48 h after transfection with siRNA using MTT assay. (**C**) BON-1 cell cycle analysis 48 h after siRNA transfection. (**D**–**F**) Expression of CCND1, CCND2 and CDK4 in cells transfected with siRNA for 48 h. Results are the mean ± S.E.M., obtained from at least *n*=4.

Next, we examined the effects of TRPV6 down-regulation on BON-1 cell proliferation. As shown in Figures 3(A) and 3(B), down-regulation of TRPV6 protein production attenuated BON-1 cell proliferation. To further confirm the role of TRPV6 in controlling BON-1 cell growth, we analysed cell cycle in nt and TRPV6 siRNA-transfected cells. As shown in Figure 3(C), the number of cells in G_1_-phase increased after down-regulation of TRPV6. In contrast, a decreased number of cells in S- and G_2_-M phases was detected after experimental reduction of TRPV6 protein production.

Next, we evaluated the effects of TRPV6 down-regulation on cyclin D1 (CCND1), cyclin D2 (CCND2) and cyclin-dependent kinase 4 (CDK4). Importantly, these genes are relevant for the regulation of calcium-dependent cell proliferation [15,21]. We found that TRPV6 siRNA-transfected cells had lower expression of CCND1 and CDK4, whereas expression of CCND2 remained stable as compared with nt siRNA-transfected cells (Figures 3D–3F). Overall, these results indicate that TRPV6 stimulates BON-1 cell proliferation.

### NFAT modulates BON-1 cell growth and viability

**Figure 4:**
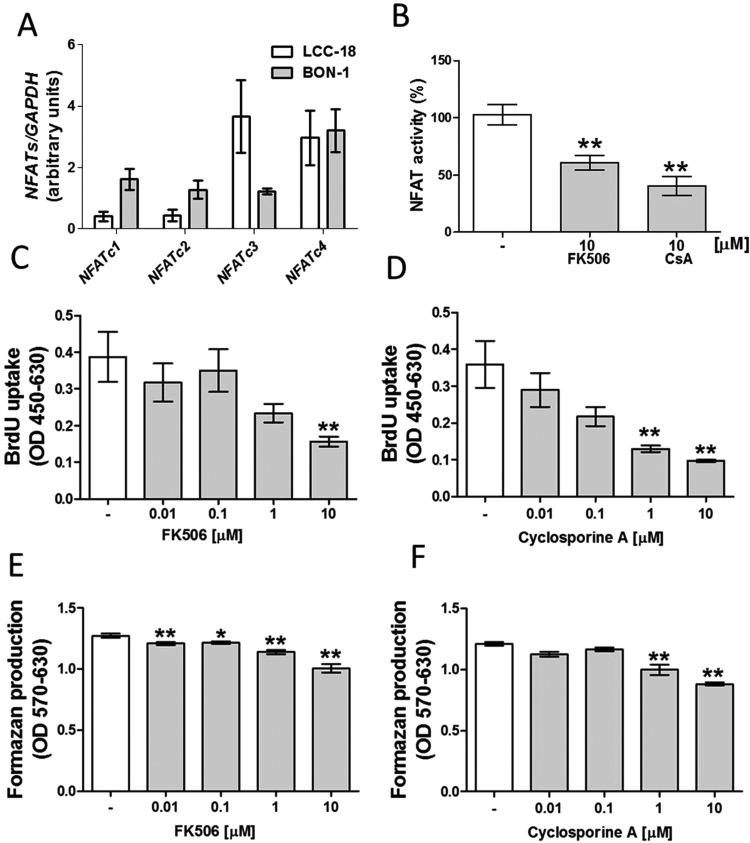
Effects of NFAT suppression on BON-1 cells proliferation (**A**) Expression of NFATs in BON-1 and LCC-18 cells. (**B**) NFAT activity in BON-1 cells treated with 10 μM FK506 or 10 μM CsA for 24 h. BON-1 cell proliferation treated with FK506 (**C**) or CsA (**D**) for 24 h. The number of viable BON-1 cells assed after 24 incubation in the presence of FK506 (**E**) or CsA (**F**). Results are the mean ± S.E.M., obtained from at least *n*=4.

Previous studies indicated that TRPV6 modulates proliferation of LNCaP human prostate adenocarcinoma or INS-1E cells via NFAT-dependent mechanisms [6,15]. As demonstrated in Figure 4(A), BON-1 and LCC-18 cells express all calcium sensitive NFAT isoforms. Since the role of NFAT at controlling NET cell proliferation is unknown, we assessed whether two different well-characterized pharmacological inhibitors of NFAT activity (cyclosporine A and FK506) [22] can influence BON-1 cell growth. As expected, both cyclosporine as well as FK506 attenuated NFAT activity (Figure 4B). Furthermore, both NFAT inhibitors reduced BON-1 cell proliferation in a dose-dependent fashion (Figures 4C–4F). In summary, these data show that NFAT stimulates BON-1 cell growth.

### TRPV6 modulates NFAT activity but not NFAT expression

**Figure 5:**
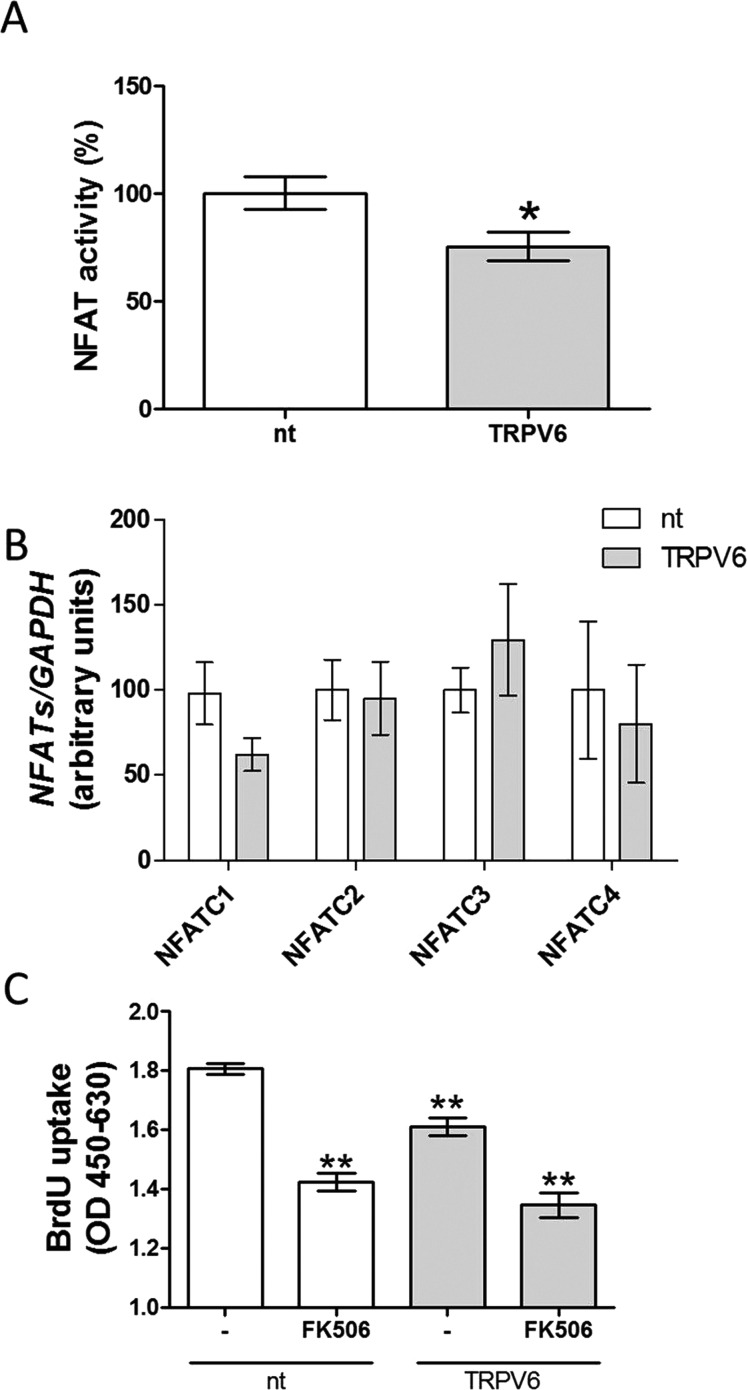
NFAT activity and expression in BON-1 cells with down-regulated TRPV6 NFAT activity (**A**) and expression (**B**) evaluated in cells transfected either with nt (white bars) or TRPV6 siRNA (grey bars) for 48 h. (**C**) Effects of 24 h treatment with FK506 on BON-1 cells transfected with nt (white bars) or TRPV6 siRNA (grey bars) for 48 h. Results are the mean ± S.E.M. (*n*=4).

TRPV6 can modulate NFAT activity in Caco-2 (human epithelial colorectal adenocarcinoma), LNCaP and INS-1E cells [6,15,23]. Therefore, we examined the effect of TRPV6 down-regulation on NFAT activity in BON-1 cells. As a result, TRPV6 siRNA-transfected BON-1 cells had decreased NFAT activity as compared with nt siRNA-transfected cells (Figure 5A). In contrast, expression of NFATs was not affected by TRPV6 siRNA down-regulation (Figure 5B). To confirm whether TRPV6 modulates BON-1 cell proliferation via NFAT-dependent mechanism, we investigated whether pharmacological blockade of NFAT activity in cells with down-regulated TRPV6 protein production has an additive effect on inhibition of BON-1 cell proliferation. FK 506 failed to further suppress BON-1 proliferation in cells with down-regulated TRPV6. These results demonstrate that TRPV6 modulates NET cell proliferation by affecting NFAT activity.

## DISCUSSION

In the present study, we detected the presence of TRPV6 channel in human pancreatic BON-1 NET cells for the first time. Furthermore, we demonstrated that TRPV6 controls calcium homoeostasis as well as proliferation of pancreatic BON-1 NET cells.

Recently, we and others showed that pancreatic NETs express several members TRP channels such as TRPV1 as well as TRPM8 channels [4,24]. Both TRPs regulate intracellular calcium concentration and various secretory activities of NET cells.

In the present study, we specifically showed that different human pancreatic NET cell lines (BON-1, QGP-1) as well as colonic LCC18 NET cells express TRPV6 at mRNA and protein levels, however at striking differences (Figure 1). Among pancreatic NET cell lines BON-1 showed a high TRPV6 expression, whereas QGP-1 poorly expressed TRPV6. Using siRNA, we also showed that suppression of TRPV6 protein production in BON-1 cells is associated with a decreased calcium response patterns, which is in accordance with previously published protocol for detection of TRPV6 channel activity [6] (Figure 2). In our previous study using NET BON-1 cells and the same protocol, we detected similar Ca^2+^ response patterns [4]. These response patterns were clearly suppressed by different TRP channel blockers indicating TRP channel activity in BON-1 cells [4]. Finally, the link between TRPV6 and calcium homoeostasis is in agreement with previous studies performed in other neoplasms such as breast, prostate cancer cells or insulinoma cells [6,15,25].

Several studies showed that TRPV6 modulates cell proliferation via Ca^2+^-dependent mechanism. In the present study, we found that suppression of TRPV6 protein production decreases BON-1 cell growth by approximately 30% and leads to declined CCND1 and CDK4 expression, without affecting CCND2 (Figure 3). Both CCND1 and CDK4 are important for cell cycle regulation [15,21,26]. Importantly, we assessed NET cell proliferation in the presence of 10% serum (FCS) to better reflect the physiological conditions. Therefore, our experimental data strongly suggest that TRPV6 is a potent regulator of BON-1 cells proliferation, even in the presence of serum.

There is convincing evidence indicating that TRPV6 modulates NFAT activity [6,15]. Transcription factors of the NFAT family (NFATs), initially described in immune cells, are required for calcium-dependent immune responses [27]. Moreover, NFATs were also detected in other tissues including heart, brain and pancreas [21,27]. Although it was already reported that NFAT expression and activity are required for pancreatic β or insulinoma cells growth [15,21], nothing is known neither about the expression, nor potential roles of NFATs in controlling NETs growth, and death. This transcription factor family is composed of five members termed as NFATc1, NFATc2, NFATc3, NFATc4 and NFAT5. First four members (NFATc1-NFATc4) are modulated via calcium signalling [28]. Therefore, we initially investigated whether calcium-regulated NFATs (NFATc1-NFATc4) are expressed in our NET cells, at all. As a result, we identified all of them in tested NET cell lines (Figure 4). Notably, previous studies showed that NFAT pathway confers promitogenic properties of TRPV6 in LNCaP or INS-1E cells [6,15]. In INS-1E cells, suppression of TRPV6 protein production or NFAT activity was associated with reduced expression of Ca^2+^-regulated genes, such as CCND2 and CDK4. Concordantly, it was earlier demonstrated that down-regulation of TRPV6 in INS-1E cells was accompanied by attenuated cell proliferation and viability [15]. In the present study, we assessed the consequences of pharmacological suppression of NFAT activity in regulating BON-1 cells growth. Both FK506 and cyclosporine A (CsA) lowered NFAT activity (Figure 4). Furthermore, BON-1 cell proliferation was reduced in response to cyclosporine or FK506 treatment. Nevertheless, it must be pointed out that cyclosporine exerts other activities such as inhibition of mitochondrial permeability transition pore opening [29].

This data suggest that cyclosporine may also modulate cell proliferation independently of NFAT inhibition, e.g. by affecting mitochondrial functions. However, a vast majority of studies showed that inhibition of mitochondrial permeability transition pore opening is relevant in the context of CsA-induced cytoprotection. For instance, CsA can protect β-cells from death induced by high glucose or promote cardioprotection by inhibiting mitochondrial permeability transition [30,31]. CsA can also increase retinal ganglion cell survival by preventing mitochondrial alteration in ischemic injury [32].

Additional novel finding in our study is that NFAT activity decreased after down-regulation of TRPV6 protein in BON-1 cells (Figure 5). This corresponds to observations in a prostate cancer LNCaP cell line or insulin secreting INS-1E cell line [6,15]. Importantly, we observed that pharmacological blockade of NFAT in cells with down-regulated TRPV6 protein had no additional antiproliferative activity in BON-1 cells. NFAT activity is presumably modulated by changes in intracellular calcium levels [33]. There is strong evidence that extracellular Ca^2+^ ions are required to activate NFAT. For example depletion of extracellular Ca^2+^ causes a suppression of transcription activity of NFAT in neuronal PC12 cells [34]. Thus, since we observed that cells with TRPV6 down-regulation had a low NFAT activity, these results indicate that TRPV6 controls intracellular Ca^2+^ levels by modulating calcium transport from extracellular environment. The relationship between TRPV6, intracellular Ca^2+^ levels and NFAT signalling is well-supported by literature [6,15,23].

Overall, these data indicate that the active NFAT is essential to maintain the growth of NETs cells and allows us to suggest that TRPV6 may control BON-1 cells growth via NFAT-dependent mechanism.

Overall, our results show a functional link between TRPV6 and NFAT activity and emphasize the relevance of this interaction at maintaining BON-1 NET cell growth. One of the limitations of our study is the exclusive use of NET cell lines instead of primary NET cells. Regarding other Ca^2+^ channels, however, we could show similar electrophysiological characteristics between several NET cell lines and corresponding primary NET cells [4,24,35]. Therefore, we suggest that particularly the aforementioned BON-1 cell line is a valid surrogate NET cell model to characterize Ca^2+^ channels as well as TRPV6. Further studies are required to confirm the role of TRPV6 at modulating calcium-dependent cell growth. Furthermore, despite conduction of our experiments in the presence of 10% serum, our study fails to identify the endogenous stimuli of TRPV6 activity in NETs. However, this is not the focus of our study.

Moreover, it remains a matter of debate whether TRPV6 is constitutively active at physiological conditions. Several studies suggested that TRPV6 is characterized by constitutively activated Ca^2+^ permeability at physiological membrane potentials [36]. Other studies indicated that TRPV6 activity is modulated by changes in intracellular and extracellular Ca^2+^ concentrations or plasma membrane depolarization (extensively studied by Bodding et al. [37]). Notably, there is evidence indicating that TRPV6-mediated calcium influx can be potentiated by 17β-oestradiol [38]. Importantly, 17β-oestradiol was demonstrated to increase breast cancer cell proliferation [39] which show extremely high density of TRPV6 expression [11]. Therefore, it is possible that 17β-oestradiol may play a role in regulating cell growth, also in pancreatic NET cells.

Further studies using primary NETs are required to evaluate the potential clinical relevance of our results. For example, a link between TRPV6 and various growth factors relevant in NETs proliferation should be assessed in the future.

In summary, our study shows for the first time that TRPV6 is expressed in pancreatic NETs, where it modulates intracellular calcium concentration. Furthermore, we show that suppression of TRPV6 protein production is associated with impaired pancreatic NET cell growth.

## Abbreviations

BrdU: bromodeoxyuridine
CCND1: cyclin D1
CCND2: cyclin D2
CDK4: cyclin-dependent kinase 4
CsA: cyclosporine A
NET: neuroendocrine tumour
NFAT: nuclear factor of activated T-cells
nt: non-targeting siRNA
TRP: transient receptor potential
TRPV6: transient receptor potential cation channel vanilloid subfamily member 6
